# Computerised therapy for depression with clinician vs. assistant and brief vs. extended phone support: study protocol for a randomised controlled trial

**DOI:** 10.1186/1745-6215-13-151

**Published:** 2012-08-27

**Authors:** Lina Gega, Louise Swift, Garry Barton, Gillian Todd, Nesta Reeve, Kelly Bird, Richard Holland, Amanda Howe, Jon Wilson, Jo Molle

**Affiliations:** 1 Norwich Medical School, University of East Anglia, Norwich NR4 7TJ, UK; 2 Norfolk & Suffolk NHS Foundation Trust (NSFT), Hellesdon Hospital, Norwich NR6 5BE, UK; 3 NHS Norfolk, Lakeside 400, Old Chapel Way, Broadland Business Park, Norwich NR7 OWG, UK

**Keywords:** Cognitive behaviour therapy, Internet, Guided self-help

## Abstract

**Background:**

Computerised cognitive behaviour therapy (cCBT) involves standardised, automated, interactive self-help programmes delivered via a computer. Randomised controlled trials (RCTs) and observational studies have shown than cCBT reduces depressive symptoms as much as face-to-face therapy and more than waiting lists or treatment as usual. cCBT’s efficacy and acceptability may be influenced by the “human” support offered as an adjunct to it, which can vary in duration and can be offered by people with different levels of training and expertise.

**Methods/design:**

This is a two-by-two factorial RCT investigating the effectiveness, cost-effectiveness and acceptability of cCBT supplemented with 12 weekly phone support sessions are either brief (5–10 min) or extended (20–30 min) and are offered by either an expert clinician or an assistant with no clinical training. Adults with non-suicidal depression in primary care can self-refer into the study by completing and posting to the research team a standardised questionnaire. Following an assessment interview, eligible referrals have access to an 8-session cCBT programme called *Beating the Blues* and are randomised to one of four types of support: brief-assistant, extended-assistant, brief-clinician or extended-clinician.

A sample size of 35 per group (total 140) is sufficient to detect a moderate effect size with 90% power on our primary outcome measure (Work and Social Adjustment Scale); assuming a 30% attrition rate, 200 patients will be randomised. Secondary outcome measures include the Beck Depression and Anxiety Inventories and the PHQ-9 and GAD-7. Data on clinical outcomes, treatment usage and patient experiences are collected in three ways: by post via self-report questionnaires at week 0 (randomisation) and at weeks 12 and 24 post-randomisation; electronically by the cCBT system every time patients log-in; by phone during assessments, support sessions and exit interviews.

**Discussion:**

The study’s factorial design increases its efficiency by allowing the concurrent investigation of two types of adjunct support for cCBT with a single sample of participants. Difficulties in recruitment, uptake and retention of participants are anticipated because of the nature of the targeted clinical problem (depression impairs motivation) and of the studied interventions (lack of face-to-face contact because referrals, assessments, interventions and data collection are completed by phone, computer or post).

**Trial registration:**

Current Controlled Trials ISRCTN98677176

## Background

Depression is one of the most prevalent and burdensome health and social problems world-wide [1]. Access to evidence-based psychological interventions, especially cognitive behaviour therapy (CBT), is restricted because of scarce healthcare resources and too few trained therapists [2]. Computerised cognitive behaviour therapy (cCBT) can be used as an alternative, or as a primer, to traditional face-to-face CBT for mild-to-moderate depression within a stepped care model [3].

A recent meta-review [4] supported the efficacy of cCBT for depression, drawing on ten systematic reviews and numerous randomised controlled trials (RCTs) that had demonstrated that cCBT for depression can be more effective than treatment as usual [5,6] or than a waiting list control [7,8], and as effective as therapist-delivered face-to-face CBT [9,10]. cCBT is generally an acceptable intervention to patients [11,12]; however, its uptake is low and the drop-out rate can be high [13-15]. This is common for e-health interventions [16] and more so for open access websites and observational studies rather than for controlled trials [17,18].

The efficacy and acceptability of cCBT may be influenced by whether, and how much, human support is offered as an adjunct to it. A meta-analysis [19,20] found that Internet- delivered CBT was four times more effective when delivered with a supportive online therapist contact. Two further meta-analyses [10,20] indicated that the longer the therapist input, the better the clinical outcomes with cCBT. A recently published RCT [7] found marginally better outcomes for depression with cCBT supplemented by weekly email therapist support compared to unguided cCBT. Two other RCTs on social phobia found that cCBT with therapist guidance by email was superior to unguided cCBT [21] and an Internet-accessed CBT self-help manual had better outcomes when supplemented by an online discussion group [22]. Also, users of a self-help book for bulimia, albeit not computerised, improved more if they had some personalised guidance rather than none [23,24].

The relationship between adjunct support and improved adherence or outcomes with cCBT is not always supported in the literature. Having three face-to-face support sessions with a therapist as an adjunct to cCBT for bulimia nervosa did not enhance adherence or outcomes compared to cCBT with minimal guidance [25]. Also, weekly telephone tracking for cCBT for depression did not have an advantage over standalone cCBT [26]. Finally, more frequent therapist contact via email (three times vs. once per week) did not improve outcomes and adherence with cCBT for panic [27]. There is a need for further research to investigate whether keeping support sessions for cCBT for depression as brief as possible can save staff time without significantly compromising outcomes and acceptability, or whether offering longer support sessions can yield greater patient improvement or better completion rates.

Some cCBT studies [21] use therapists who, whilst more expensive, may satisfy patients’ expectations and needs better than non-experts. Other studies use less experienced support staff, such as an administrator [28,29], lay counsellors [26] or psychology students [22], who are easier to find and less costly to employ, but may be less effective or less acceptable, or may have hidden costs (for example, increased patient use of other services). Two studies [28,29] directly compared cCBT with phone or email support from a therapist vs. an assistant and found similar symptom improvement in people with depression and generalized anxiety disorder (GAD) between the two groups. No studies have so far investigated the cost-effectiveness or explored patients’ experiences of receiving therapist vs. assistant support as an adjunct to cCBT.

This paper describes the protocol for a factorial RCT whose primary aim is to compare the clinical effectiveness, cost-effectiveness and acceptability of cCBT with two different types of support offered as an adjunct to it: brief vs. extended, and clinician vs. assistant support. To this end the study has the following primary objectives:

1. To compare clinical outcomes (depression and anxiety symptoms, functioning and quality of life) between brief and extended support for cCBT and between clinician and assistant support for cCBT.

2. To assess the cost-effectiveness of cCBT with brief vs. extended support and with clinician vs. assistant support and to estimate the cost for the National Health Service (NHS} of the population-wide implementation of the most cost-effective option.

3. To report and compare acceptability of cCBT (attrition, treatment usage and patient experiences) between brief vs. extended support and between clinician vs. assistant support.

The study also aims to evaluate the process of recruiting and screening patients with depression for cCBT, to explore effect modifiers (specifically severe vs. mild/moderate depression or anxiety, and referrals from general practitioners (GPs) vs. mental health professionals) and predictors of cCBT usage and outcomes, to indicate the most appropriate measures for assessing clinical outcomes with cCBT, and to compare the computer and the support person with respect to creating and maintaining a therapeutic alliance with the patient. To this end, the study has the following secondary objectives:

1. To compare the characteristics of referrals for cCBT who take up the offer for assessment and treatment with the characteristics of referrals who drop out before randomisation (refusals).

2. To assess whether a clinician or an assistant can reliably predict patient suitability for cCBT by reviewing patients’ written responses to a standardised questionnaire.

3. To explore whether outcomes and satisfaction with different levels of support for cCBT vary for certain sub-groups of patients (severe vs. mild/moderate depression or anxiety and referrals from GPs vs. mental health professionals).

4. To investigate whether patient characteristics and treatment group predicts clinical outcomes, number of cCBT sessions completed, and patient satisfaction.

5. To assess the similarity of various standardised self-report questionnaires for the assessment of depression, anxiety and functioning and quality of life.

6. To compare and assess the similarity of alliance ratings and explore patients’ experiences using a computerised self-help system vs. speaking to a support person on the telephone.

7. To identify common themes and key differences in the content and style of brief vs. extended support and of clinician vs. assistant support.

## Methods/design

### Design

This is a two-by-two factorial RCT. Patients are randomly allocated to an eight-session cCBT programme supported either by a clinician or by an assistant, each offering either brief or extended phone support. The four randomly-created groups are: brief-clinician, brief-assistant, extended-clinician or extended-assistant support for cCBT. The primary follow-up point is at 3 months post-randomisation and the secondary follow-up point is at 6 months post-randomisation.

### Sample

Patients are eligible for the trial if: their primary problem is non-suicidal, non-psychotic, unipolar mood disorder, such as depression or dysthymia or mixed anxiety and depressive disorder based on ICD-10 criteria [30]; they sign a written consent form for screening and for treatment; they are registered with a GP within the local area and they are at least 18 years old. There is no upper cut-off for severity of depression or anxiety symptoms and participants can have comorbid physical and mental health problems and receive concurrent treatments. Referrals are only excluded if they have: high current risk of suicide (current suicide plans and intent to kill themselves); current psychotic symptoms (due to schizophrenia or bipolar affective disorder or severe depression with psychotic features); current substance abuse/misuse (severe dependency on alcohol, illicit drugs or tranquilisers) or cognitive impairment that impedes the use of a computer.

### Recruitment

Health professionals working in primary care, or in the voluntary sector, signpost patients to the study by handing them an envelope containing a patient information sheet, a consent form and a self-referral questionnaire. People who find out about the study through public advertisement can also contact the research team directly by telephone or email, or can download the study entry documents from a designated website. Patients are encouraged to email or telephone the research team before completing and posting the self-referral questionnaire and consent form, should they have any questions or if they wish to discuss something confidential.

### Assessment

On receiving a completed self-referral questionnaire and consent form, the study’s research associate (JM) assigns a unique trial identifier number to the patient and copies their self-referral questionnaire twice after masking any identifiable patient details. The anonymised copies of the self-referral questionnaire are distributed to the study’s assistant (KB) and to two expert clinicians (NR, GT). They all independently read the self-referral questionnaire, and record their judgment on the patient’s diagnosis and suitability for cCBT. The research associate (JM) invites the patient for a brief (15 to 30 minutes) telephone interview to determine their eligibility for study entry and to discuss any questions they may have about the study. If a patient is not eligible, the research associate (JM) discusses this with them, suggests alternative avenues for help, and records the reasons for ineligibility in a web-based database.

### Treatment allocation

**Figure 1:**
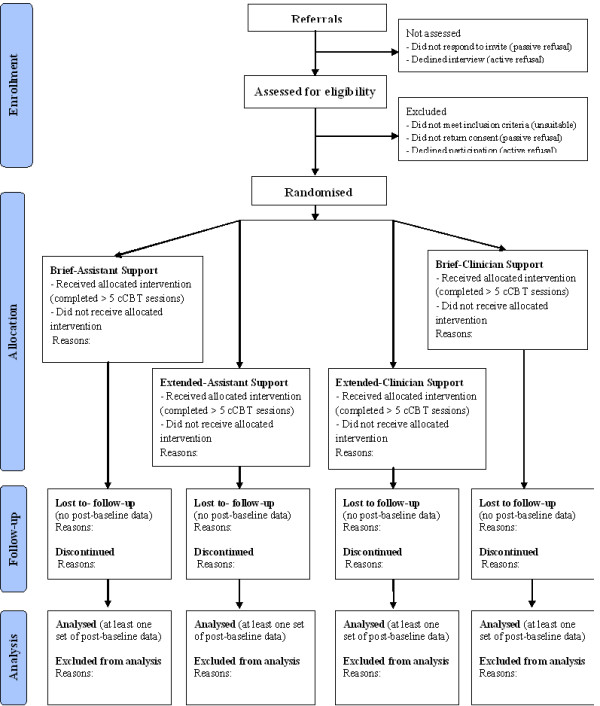
CONSORT* diagram of participant flow (*according to Schulz KF, AltmanDG, Moher D, the CONSORT Group, 2010).

Patients eligible for trial entry receive a treatment consent form and a set of blank standardised self-report questionnaires (baseline assessments) to complete and post to the research team in a stamped addressed envelope. On receiving the completed treatment consent form and baseline assessments, the research associate (JM) randomises patients into one of the four study intervention groups using a web-based system set up by the Clinical Research Trials Unit (CRTU) of the Norfolk & Norwich University Hospitals (NNUH) Trust. The system follows a block-randomisation: for every 32 referrals, it ensures that eight patients are assigned to each of the four arms of the trial so that the clinician and assistant who offer phone support to patients have similar workload distribution over time. Figure 1 is a CONSORT diagram [31] of the randomisation and flow of participants through the study. 

### Interventions

After randomisation, the research associate (JM) emails or telephones the patient with an access code for logging into the cCBT system and with the details of the person who will be supporting them. Patients are registered with the system as active participants once they log in for the first time and choose their own password. The support person emails or telephones the patient within a week of randomiation to introduce themselves and schedule their support sessions. All patients are offered an intervention with two components, cCBT and some type of adjunct telephone support.

### cCBT

The computerised cCBT system used in this study is Beating the Blues (BtB) marketed by Ultrasis Ltd based in London, UK. This was chosen because at the time of the study set-up it was the cCBT system recommended in the UK by the National Institute for Clinical Excellence (NICE) [3] and the Department of Health (DH) [32] as an effective and cost-effective treatment method for mild-moderate depression in primary care. During eight 50-minute sessions, BtB uses text, videos, voice instructions, diagrams and pictures to teach patients how to identify unhelpful thoughts and come up with helpful alternatives, and how to perform activity scheduling, problem-solving and other relevant homework tasks between their computer sessions. Each computer session builds on the one before and its completion triggers printing of a progress report.

Most patients use the computerised therapy system from home; those who do not have access to a computer, or who wish to use the system outside their home environment, can access the cCBT system in public places such as libraries, community centres or Internet cafes and are reimbursed for their expenses. Patients go through BtB at their own pace, but it is suggested that they cover a module per week, allowing enough time in-between sessions for homework (for example, rehearsing practical tasks or keeping diaries), with the aim of completing the whole programme within 12 weeks.

#### Telephone support

Individual support is given as a scheduled, telephone call on average once a week or fortnight (depending on how fast the patient goes through the programme) for 3 months or longer, and up to 6 months if needed. Support sessions are either brief (5 to 10 minutes per call) or extended (20 to 30 minutes per call). Patients can receive up to 12 telephone calls over 12 to 24 weeks, so they have a maximum of 1 to 2 hrs of brief support or 4 to 6 hrs of extended support throughout using the cCBT system. *Ad-hoc* contact is also available, in case patients raise risk issues or get stuck with the system.

Both brief and extended support sessions focus on monitoring patient progress and mental state as necessary. Extended support sessions offer additional therapy advice by elaborating on some of the treatment techniques described in the cCBT system (for example, to help patients identify thinking errors and alternative beliefs, problem-solving difficulties with doing homework, etcetera). Support is not standardised, so that it can be tailored to individual patients’ needs and preferences.

Support sessions are offered either by a clinician (JT), a PhD nurse with CBT clinical qualifications and 20 years clinical experience, or by an assistant (KB), a graduate psychologist with no clinical qualifications but with 2 years experience of working with people with depression and anxiety in a voluntary organisation. Both members of staff receive individual telephone or face-to-face clinical supervision from a senior CBT-trained Clinical Psychologist (NR) and research supervision from the Chief Investigator (LG).

### Data collection

Baseline demographic and clinical characteristics are obtained by the self-referral questionnaire which is completed by patients and posted to the research team before randomization. The self-referral questionnaire has been suitably adapted for the purpose of this study from its previous validated version [33]. The questionnaire has nine sections which elicit quantitative and narrative data about patients’ symptoms, computer literacy, lifestyle, past and present treatments including medication, and therapy goals and preferences for therapist-delivered interventions vs. computerised self-help.

Data on clinical outcomes, resource use and patient experiences are collected in two ways: both by post and electronically. Postal questionnaires are sent to patients at baseline (randomization) and then at weeks 12 (primary end-point) and 24 (follow-up) post-randomization with instructions to complete them and return to the research team. Electronic data are collected automatically by the cCBT system during each cCBT session. The timing of electronic data collection is recorded by the cCBT system but it varies according to the frequency with which patients log into it.

Narrative data are also collected during a 15 to 30 minute assessment with the patient pre-randomization, as part of support sessions by the clinician and the assistant, and during a 15 minute telephone interview, which asks about patient views and experiences of using a computerised self-help system and of having telephone support at 12-weeks post-randomization, by the study’s research associate (JM).

Below we give a detailed description of individual data collection tools and justification for their inclusion in the study. All standardised measures are self-reported and are collected independently of the staff proving telephone support; there are no observer-rated outcomes so there is no need for blinding.

#### Clinical outcomes

Work and social adjustment scale (WSAS) [34,35]: this is a 5-item self-report questionnaire that assesses impairment of everyday functioning with scores of 0 to 40 (lower scores denote less impairment). Each item asks the patient to rate how much a particular area of their life is affected by their problem (work, social activities, private leisure activities, relationships) on a 9-point scale, where 0 represents ‘not at all’ and 8 represents ‘very severely’. This is a brief and well-validated measure that has previously been used in many studies and is part of routine data collection in the NHS Improving Access to Psychological Therapies (IAPT) Programme [36].

We chose a generic measure of functioning instead of a symptom-specific measure for our primary outcome for three reasons: a) improved functioning is a key objective of primary care mental health services with the view of helping people return to work [37]; b) people with depression and mixed anxiety are a heterogeneous group and c) we can establish cost-effectiveness measuring effects of functioning with both the WSAS, commonly used by clinicians, and the EQ-5D, commonly used by health economists.

Beck depression inventory (BDI) [38]: this is a 21-item questionnaire that measures presence and severity of depression. Each item relates to a specific symptom of depression and includes four statements corresponding to scores 0, 1, 2 or 3 (higher scores, greater severity). Patients choose one of the four statements to describe the extent to which they were troubled by each symptom over the previous week. The items are then summed to obtain a total score that can range from 0 to 63 (higher total scores indicate more severe depression). Standard cut-offs are: 0 to 9, no depression; 10 to 18, mild-moderate depression; 19 to 29, moderate-severe depression and 30 to 63, severe depression.

Beck anxiety inventory (BAI) [39]: this is a 21-item self-report questionnaire that assesses the presence and severity of anxiety. Each of its items corresponds to a common symptom of anxiety, which patients rate based on how much they were troubled by that symptom over the previous week (0, not at all; 1, mildly troubled; 2, moderately troubled and 3, severely troubled). The items are then summed to obtain a total score that can range from 0 to 63 (higher scores, greater anxiety). Common cut-off points are: 0 to 21, low anxiety; 22 to 35, moderate anxiety and over 36, severe anxiety.

Health-related quality of life - EQ-5D [40]: this is a five-domain measure of health-related quality of life covering mobility, self-care, usual activities, pain/discomfort and anxiety/depression. Each domain has three levels and patients tick the statement that best describes their state of health at the time (no problems, 1; some/moderate problems, 2 and severe/extreme problems, 3). Responses to these five domains are converted into one of 243 different health state descriptions, which range between no problems in all five domains (11111) and severe/extreme problems in all five domains (33333). Patients also rate their overall impression of how good or bad their health state is on the day of completion using a separate scale of 0 to 100 (0 , worst imaginable health state; 100, best imaginable health state). We have selected the EQ-5D because it is commonly used to estimate quality-adjusted life years (QALYs) for the economic evaluation of health interventions [41] and allows comparisons of the cost-effectiveness of different interventions across different conditions.

Patient health questionnaire (PHQ) - depression-9 item (PHQ-9) [42] and generalised anxiety disorder-7 item (GAD-7) [43]: the PHQ-9 and GAD-7 are brief validated scales to screen for the presence and severity of depression and generalised anxiety respectively, in primary care. Each of the 9 items of PHQ-9 and of the 7 items of GAD-7 describes a common symptom of depression or generalised anxiety respectively, which patients rate on a scale of 0 to 3 according to how often they have been troubled by this symptom in the previous 2 weeks (0, not at all; 1, for several days; 2, for more than half the days; 3, nearly every day). Standard cut-offs for PHQ-9 are 0 to 4, no depression; 5 to 9, mild depression; 10 to 14, moderate depression; 15 to 19 moderately severe depression, 20 to 27 severe depression. For GAD-7 the cut-offs are 0 to 4, no anxiety; 5 to 10, mild anxiety; 11 to 15, moderate anxiety; 15 to 21, severe anxiety.

The PHQ-9 and GAD-7 are subsidiary clinical outcome measures collected by post at 12 and 24 weeks post-randomization, and electronically every time patients log into the cCBT system. We have included them in the study because they are routinely used in the NHS to screen for depression and anxiety in primary care and to evaluate the effectiveness of psychological therapies nationwide [36]. These will allow us to compare our study findings with published national outcomes on cCBT and related interventions [44,45]. We will also test whether the PHQ-9 and GAD-7 can be as sensitive as the BDI & BAI in measuring depression and anxiety respectively but less cumbersome for patients to complete.

#### Single-item anxiety and depression scores

The cCBT system asks patients to rate two questions at the beginning of their session every time they log-into the cCBT system (8 times). These are: ‘How anxious or stressed have you felt in the past week?’ where 0 represents not at all and 8 represents extremely anxious), and ‘How depressed have you felt in the past week?’ where 0 represents not at all and 8 represents extremely depressed,.

#### Suicide

The cCBT system automatically asks questions about the presence & frequency of suicide thoughts and about suicide intent at the beginning of each session every time patients log-into the cCBT system (8 times). Patients answer the following questions: ‘Have you had any thoughts about suicide in the last week?’ (yes/no). If yes, ‘How often have you thought about ending your life in the last week?’ (once, twice, three times, more than three times), and ‘How seriously have you planned to carry it out?’ (0, not very seriously; 8, very seriously).

#### Individual problem scores

The patient describes up to three problems during their first cCBT session every time they log in to the cCBT system at the beginning of their session (8 times) and then they rate the level of distress that each problem causes for them (0, no distress; 8, extreme distress).

#### Patient experiences

#### Computer-patient alliance scale (C-PAS) and therapist-patient alliance scale (T-PAS)

These are two 15-item questionnaires that measure patients’ experiences of using computerised self-help and of having individualised support. Each item asks the patient to rate the statement that best describes their experience with either cCBT or with the support they received, on a 9-point scale, where 0 represents ‘not at all’, and 8 represents ‘extremely’). The two scales are matched item-by-item to allow comparisons of the same therapeutic elements between using a computer vs. speaking to a person (for example, ‘The therapist has contributed to improving my problems.’ vs. ‘The computer system has contributed to improving my problems.’).

These are bespoke scales developed by the Chief Investigator (LG) to assess therapy processes and patient experiences in relation to this study. Existing standardised measures, such as the Agnew relationship measure [46] or the working alliance inventory [47], are designed for conventional face-to-face psychotherapy and include many items that are not relevant or did not address the objectives of this study.

#### Patient experience interview (PEI)

The interview follows a structured guide with specific questions and prompts under four headings: a) general experiences and perspectives of treatment; b) hindering, unhelpful, negative or disappointing aspects of treatment; c) productive, helpful, positive or encouraging aspects of treatment; d) suggestions and additional comments.

#### cCBT system usefulness, relevance and ease of use

The cCBT system automatically asks three questions at the end of each cCBT session (8 times) about patient experiences of using the system. These are; ‘Looking at session x: How useful was it?’ (on a scale of 0 to 8, where 0 represents not at all useful, and 8 represents very useful); ‘Was it relevant to your problems? (on a scale of 0 to 8, where 0 represents not at all relevant, and 8 represents very relevant) and ‘How easy was it to follow?’ (on a scale of 0 to 8, where 0 represents very difficult, and 8 represents very easy).

#### Resource use

#### Resource use questionnaire (RUQ)

The RUO is a standardised measure developed for this project to estimate which resources and services patients have been using in relation to their depression before, during and after cCBT. The questionnaire has 10 sections that elicit information about patient contact with healthcare professionals, use of other agencies and services, medication and help from friends and family.

#### Therapist session records

Support staff maintain detailed electronic records of the content of their sessions with individual patients and their actual duration using a stop-watch; these are kept in a password-protected web-based database at the UEA. A random selection of the support sessions is taped each time with the patient’s agreement. This is to ensure session fidelity to the study protocol and to allow for qualitative comparisons in the style and content of support between the clinician and the assistant. The support staff also keep written records of the duration and content of their supervision sessions with the study’s clinical supervisor (NR).

### Sample size

Sample size calculations are based on a between-group comparison of post-treatment scores at 12-weeks follow-up post-randomisation on our primary outcome measure (WSAS), assuming a significance level of 0.05 with a standard deviation of 8.5 for that measure; this standard deviation was taken from Proudfoot *et al*. [6], whose study used the same intervention, outcome measure and follow-up interval.

A sample size of 35 per group (total 140 patients) is sufficient to detect main effects (brief vs. extended, and clinician vs. assistant) of 0.477 standard deviations (about 4.1 points) in our primary outcome measure with 80% power, or of 0.522 (about 4.4 points) in our primary outcome measures with 90% power. The estimated sample size of 140 patients with available data can detect an interaction effect (for example, the difference between brief and enhanced, compared between clinician and assistant) of about 0.961 standard deviations (about 8.2 points) on our primary outcome measure with 80% power.

Allowing for a 30% drop-out rate, we intend to randomise 50 patients in each of the four groups (total 200 randomised patients). We estimate that about a third of all screened patients may be unsuitable for the trial or refuse participation (based on a similar pragmatic trial by Marks *et al*. [48]), therefore to achieve 200 randomised patients we aim to recruit a total of 300 referrals.

Our sample size calculation and effect estimates are conservative. Our hope is that adjustment by the corresponding baseline outcome measure reduces the standard error of the effect estimates and hence increases the power for a given sample size.

### Statistical analysis

Descriptive statistics will summarise demographic and clinical features of those referred, those randomised, those who complete the intervention (at least 5 cCBT sessions) and those who complete at least one set of post-baseline outcome measures. The same variables and baseline values of the outcome measures will be compared between brief and extended support, and between clinician and assistant at baseline. Where large differences exist, these variables will be included as potential confounders. Our primary analysis will be based on the first batch of completed postal questionnaires returned by the patients. These are posted out to the patients at 12 weeks post-randomisation but the timing of receiving completed questionnaires from the patients is variable. The time of receipt is recorded and may be included as a covariate.

Linear models will be used to estimate the main effects of the two treatments. An estimate and confidence interval for the interaction term will be obtained. Corresponding adjusted estimates will be obtained adjusting for baseline only, and for baseline and the potential confounders identified at baseline. Effect sizes will be calculated using Cohen’s d [49]. The number of patients showing clinically significant and reliable change [50] will be reported where possible. C-PAS and T-PAS will be compared for each group using mixed-effects models and their similarity will be assessed using intra-class correlation.

The primary analysis will include patients in the groups to which they had been randomly assigned regardless of the actual treatment they have received. The primary analysis will include non-missing data only, but the baseline characteristics of missing and non-missing values will be compared to assess the pattern of missing data. Further, as recommended by Altman [51], sensitivity analyses will be performed by employing multiple imputation techniques. Where a few items within a scale are missing for an individual participant we will impute using that person’s scale average.

A per-protocol analysis will also be performed on the main outcomes using completers only, which we define as those patients who complete at least 5 cCBT sessions. We will compare sub-group effects by including interaction terms in appropriate linear models. We will use linear models to investigate which demographic and clinical variables predict primary outcome, session completion and patient satisfaction. Interaction terms may be included to investigate which variables modify the main effects. Secondary analysis will be carried out using the electronic data collected by the cCBT system in every session. The series of session electronic outcomes will be summarised and compared between treatment groups using repeated measures analysis.

### Qualitative analysis

Narrative data will be analysed using thematic and content analysis. Thematic analysis will identify and describe features within the textual data, whereas content analysis will look for frequency of occurrence of these features. Qualitative analysis will include repeated readings of the text to enable familiarisation with the data, noting preliminary features and patterns of interest, coding the transcripts, adding and modifying codes, and collating data relevant to existing codes until no new codes emerge [52]. The following textual data will be analysed:

1. Patients’ narrative responses on the screening questionnaire to provide an overview of commonly presenting features of depression/anxiety in primary care for patients seeking computerised therapy.

2. Clinical written records to identify staff rationale for decision-making on suitability and prognosis based on patients’ responses to a standardised questionnaire and after a telephone assessment interview.

3. Recorded sessions and written clinical records from support staff to identify prominent features in the content and delivery of different types of support.

4. Researcher records to identify reasons why patients did not take up cCBT pre-randomisation or dropped out post-randomisation.

5. Recorded exit interviews to identify aspects of cCBT or the adjunct support that patients considered relevant or helpful, or hindering or missing, and any suggestions they may have to improve the study or the intervention.

### Economic evaluation

To establish the cost-effectiveness of clinicians vs. assistants offering brief vs. extended support, we will combine the effects and costs of each of the four support modes (brief-clinician vs. brief-assistant vs. extended-clinician vs. extended-assistant support). Effects will be measured using both the WSAS and the EQ-5D. Cost will be estimated by assigning appropriate unit costs to resources used by patients (therapist contact time, cCBT use, medication use, GP consultations, in-patient and out-patient care, etcetera) before, during and after the interventions. All costs will be calculated in UK £ Sterling using both internal sources (for example, the relevant local organisations’ finance departments) and external sources (for example, Personal Social Services Research Unit (PSSRU)-health and social care costs [53]). We will estimate cost-effectiveness from the perspective of the NHS and social services according to NICE guidelines [41]) and also from a broader societal perspective (encompassing for example, patient travel costs, lost productivity and other support received).

If one support mode is shown to be less costly and more effective than all others, determining the most cost-effective support mode will be straightforward, as that support mode would dominate others. Alternatively, the incremental cost per unit of effect will be estimated for each support mode, and the resulting incremental cost-effectiveness ratio (ICER) will be assessed in relation to a range of cost-effectiveness thresholds. To characterise the level of uncertainty in decision-making based on the cost-effectiveness analysis, we will present economic data based on the cost-effectiveness acceptability curve (CEAC) and cost-effectiveness acceptability frontier (CEAF) [54].

Additionally, sub-group analysis (severe vs. mild/moderate depression or anxiety; referrals from GPs vs. mental health professionals) will be conducted in order to assess how the costs and effects of different modes of support for cCBT vary across different patient groups. Sensitivity analysis will assess the robustness of conclusions to key assumptions that are made within the economics analysis (for example, the level of payment for clinicians or assistants employed). Finally, we will estimate the budgetary impact for the NHS of the population-wide long-term implementation of the most cost-effective mode of individualised support for cCBT.

### Public & patient involvement

A focus group involving public and service user representatives was set up with help from the Patient and Public Involvement in Research (PPIRes) at the local Primary Care trust (NHS Norfolk), to discuss cCBT implementation and patient recruitment. This focus group reviewed some of the study documents (such as the patient information sheet, screening questionnaire and invitation letter) and made recommendations for their improvement and dissemination. Two of the focus group members have been participating in the study trial management group and steering committee. The same members will be actively involved in the interpretation and dissemination of the study’s findings.

### Ethical issues

Approvals were granted by a regional NHS Research Ethics Committee and the Research & Development (R&D) departments of the relevant NHS Trusts. Patient consent is obtained by post. Patients are advised to return the consent form and self-referral questionnaire by post and not by email, to protect confidentiality. Patients can withdraw from the study at any point, without providing a reason, but data collected up to their withdrawal will still be used. Patients retain cCBT access for 24 weeks even if they choose not to speak to the support staff. If a patient needs further treatment upon completion of the study, research staff can directly make a referral to the local psychological therapies service or discuss treatment options with the patient’s GP.

Patients’ research data are stored on a password-protected laptop. Information recorded on the cCBT system can only be accessed by the Chief Investigator, the study’s research administrator and the individual member of staff who offers support to each patient. The support staff do not have access to any named individual feedback about experiences of therapy. Anonymised electronic data (such as demographic information, clinical outcome ratings, extracts from patient interviews and therapist field notes) are accessible only by research and clinical staff involved in the study. Digital recordings are wiped once the transcribed data are stored safely and all identifying information is omitted from transcripts. Archive copies of postal questionnaires and transcripts will be retained for 10 years and then disposed of in accordance with the University and NHS guidelines.

## Discussion

### Methodological considerations

The factorial design of our study increases its efficiency by allowing the concurrent investigation of two different types of support for cCBT, that is, how a clinician compares to an assistant and how brief support compares to extended support, with a single sample of participants. For this, the proposed sample size of 200 randomised patients, with the aim of retaining and analysing data from 140 participants, will be adequate to detect a difference between brief and extended support (for both clinician and assistant) and between clinician and assistant (for both brief and extended support). We will estimate the interaction effect between the two different types of support, but the study is not powered to test it significance unless it is very large.

Most factorial RCTs assume that there is no interaction between their two studied interventions and the presence of strong interactions may lead to loss of power for detecting the main effects of the interventions. This study is not powered to detect an interaction between our interventions, unless this interaction is very large. Whilst a detectable interaction between the duration of support and the person giving support will be interesting from a clinical perspective and potentially informative for health service delivery, it will affect the way we interpret our main effects.

### Practical considerations

Difficulties with patient recruitment and retention in computer-based interventions are reflected in the low take-up and high attrition rates reported in the literature [13,17,55]. Moreover, this study includes people with depression, a condition which by definition impairs motivation and may make it more difficult for patients to start or continue with therapy. To maximise recruitment and take-up rates, we have kept our exclusion criteria very broad (patients could have any other concurrent treatments and there was no upper cut-off for depression severity). We have also introduced flexible ways of receiving referrals into the study (by telephone, email, text or post from various sources including GPs or any other health professionals, and by public adverts).

To prevent drop-outs and maximise cCBT usage and data completion, we have offered participants the option of accessing cCBT at home or in public places (libraries, cafes, etcetera.) and of having adjunct support or returning their questionnaires and giving us feedback via email, post or telephone. To date, all outcome measures have been received by post, all participants accessed cCBT from home and all support sessions took place by telephone with the exception of appointments or cancellations, which tend to happen via email or text.

An important point of consideration for our study is that there is no face-to-face contact with the researchers or the clinicians at any point because all referrals, assessments, interventions and data collection are completed remotely via telephone, computer or post. This lowers the research costs (for example, there is no need to rent rooms or to travel to people’s homes, or to employ extra people to allow for loss of time because of travelling) and reflects routine conditions in the UK primary care psychological therapy services, in which patient assessments and cCBT delivery are completed mostly by telephone as part of so-called step 2 interventions.

We cannot tell whether the lack of face-to-face contact may compromise take-up, completion or effectiveness of cCBT or whether the addition of face-to-face contact could have enhanced those. Face-to-face support has been shown to have better outcomes than telephone support in self-help for bulimia [23], and lack of face-to-face contact was the reason given by a third of all drop-outs from an online intervention for post-traumatic stress disorder [56]. On the other hand, telephone-delivered CBT (as a stand-alone intervention and not as support to a computer system) was found to be as effective and acceptable as face-to-face CBT for obsessive-compulsive disorder (OCD) [57] and for chronic fatigue syndrome [58].

### Potential implications

With depression being one of the most burdensome health problems worldwide, there is an increasing interest in technology-mediated interventions, such as cCBT, which can be accessed relatively easily in the community by large numbers of people. Literature suggests that personal support may be a necessary adjunct to cCBT to keep people motivated or to improve outcomes, but we do not know who should provide this support and for how long. The results of this study could indicate whether patients benefit more from extended vs. brief support or from support offered by a clinician or an assistant. The study will also indicate whether brief-assistant support may appear more economical but may compromise outcomes and include hidden costs, and whether the perceived more expensive option of extended clinician support can be offset by better clinical outcomes and greater patient satisfaction. Finally, the study can lead to recommendations as to how telephone support could be tailored to suit different patients and which mix of staff expertise and support duration could optimise clinical outcomes and patient satisfaction with cCBT within the current budget constraints of healthcare providers.

## Trial status

Patient recruitment was ongoing at the time of manuscript submission and stopped in June 2012. Data collection will continue until September 2012.

## Competing interests

The author(s) declare that they have no competing interests.

## Authors’ contributions

LG designed the trial and produced this manuscript, developed the patient screening and alliance questionnaires and the patient assessment and exit interview guides, produced the trial database specifications, and is the Chief Investigator responsible for the day-to-day management of the trial. LS is the trial statistician, has advised on the design of the study and helped with preparation of the protocol. GB drafted the health economics evaluation part of the protocol; with LG he also designed the resource use questionnaire for collection of the economic data. JT and KB offer telephone support to study participants and keep relevant electronic records. NR offers clinical supervision to the study support staff. RH advises on the trial design and quantitative methods and AH advises on the qualitative data analysis. JW advises on recruitment and service implementation issues. JM carries out patient telephone assessments and exit interviews and is responsible for data input and transcribing. NR, KB, JT, JM and LG review patient self-referral screening questionnaires. All authors read and approved the final manuscript.
